# Diagnostic accuracy of liver stiffness measurement in chronic hepatitis B patients with normal or mildly elevated alanine transaminase levels

**DOI:** 10.1038/s41598-018-23646-2

**Published:** 2018-03-27

**Authors:** Qiang Li, Liang Chen, Yu Zhou

**Affiliations:** 1 Department of liver disease, Shanghai Public Health Clinical Center, Fudan University, Shanghai, 201508 China; 2 Department of Infectious Disease, Huashan Hospital, Fudan University, Shanghai, 200040 China; 3 Department of Infectious Disease, Ruian people’s hospital, Wenzhou, Zhejiang, 325200 China

## Abstract

We aimed to evaluate the diagnostic accuracy of liver stiffness measurement (LSM) in 188 chronic hepatitis B (CHB) patients with alanine transaminase (ALT) ≤ twice the upper limit of normal (ULN). Liver fibrosis was staged using METAVIR scoring system. Define significant fibrosis as F2-F4, severe fibrosis as F3-F4, and cirrhosis as F4. To predict F2-F4, the AUROC of LSM was higher than that of APRI (0.86 *vs* 0.73, *p* = 0.001) and FIB-4 (0.86 *vs* 0.61, *p* < 0.001). To predict F4, the AUROC of LSM was also higher than that of APRI (0.93 *vs* 0.77, *p* = 0.012) and FIB-4 (0.93 *vs* 0.64, *p* < 0.001). Patients with ALT levels 1–2 ULN had higher cut-off values than patients with normal ALT levels for the diagnosis of F2-F4 (6.5 *vs* 6 kPa) and F4 (10.2 *vs* 7.8 kPa). Using cut-off values regardless of ALT levels, the diagnostic accuracy of LSM was 81% for F2-F4, and 89% for F4. Applying ALT-stratified cut-off values, the diagnostic accuracy of LSM was 82% for F2-F4, and 86% for F4. In conclusion, LSM is a reliable noninvasive test for the diagnosis of liver fibrosis. Applying ALT-stratified cut-off values did not enhance diagnostic accuracy of LSM in CHB patients with ALT ≤ 2 ULN.

## Introduction

Hepatitis B virus (HBV) infection is still a major public health burden. In China, the prevalence of hepatitis B surface antigen (HBsAg) is 9.75% in 1992, and 7.18% in 2006^1^. A study published in 2016 showed that the HBsAg positive rate was 6.0% in men aged 21–49 years in rural China^2^. Another study showed that the HBsAg positive rate was 6.1% in Northeastern China in 2016^3^. In the last decade, the prevalence of HBV in China has changed from highly endemic to intermediate endemic^2^. However, in China, the absolute number of patients with HBV infection is still large because of its vast denominator.

The assessment of the severity of liver fibrosis is important to identify patients for treatment and hepatocellular carcinoma (HCC) surveillance. Liver biopsy has traditionally been considered the reference method for evaluation of liver fibrosis. However, liver biopsy is a costly and invasive procedure, carrying potential complications. Therefore, non-invasive diagnostic method would be more acceptable to patients. Liver stiffness measurement (LSM) has been introduced as a new, non-invasive method for the diagnosis of liver fibrosis. Many studies found that LSM could predict liver fibrosis accurately in patients with chronic hepatitis C (CHC)^4–7^. In recent years, several studies have been performed to apply LSM to patients with chronic hepatitis B (CHB)^6,8–10^. However, these studies were mainly performed in European and United States, and the results cannot be extrapolated to Chinese patients with CHB. First, HBV genotype A is highly prevalent in Europe and United States, while genotypes B and C are common in China. Second, most CHB patients are HBeAg positive and high HBV DNA levels in China, while most CHB patients are HBeAg negative and low HBV DNA levels in Europe and United States^11,12^. Therefore, further studies should be performed in Chinese patients with CHB.

According to EASL clinical guidelines, patients with HBV DNA > 20,000 IU/ml and ALT > 2 ULN should start antiviral therapy regardless of the degree of fibrosis^13^. For these patients, liver fibrosis assessment may provide additional useful information, but it does not usually change the decision for treatment. In patients with ALT ≤ 2 ULN, liver fibrosis assessment should be used for decision on treatment indications. Patients with at least significant fibrosis should be treated^13^. Thus, patients with ALT ≤ 2 ULN have more needs for liver fibrosis assessment than those with ALT > 2 ULN. In this study, we aimed to: (1) assess the diagnostic accuracy of LSM in Chinese patients with CHB; (2) compare the diagnostic accuracy of LSM with serum fibrosis models (APRI and FIB-4); (3) evaluate the impact of ALT levels on LSM in patients with ALT ≤ 2 ULN.

## Material and Method

### Study population

**Figure 1:**
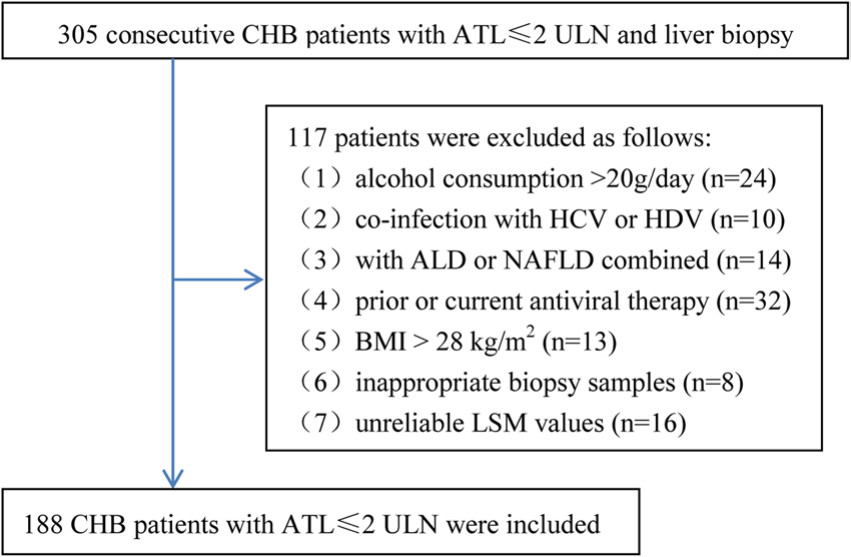
Flow diagram of the study population and reasons for exclusion. CHB, chronic hepatitis B; ALT, alanine transaminase; ULN, upper limit of normal; HCV, hepatitis C virus; HDV, hepatitis D virus; ALD, autoimmune liver disease; NAFLD, non-alcoholic fatty liver disease; BMI, body mass index; LSM, liver stiffness measurement.

A total of 305 consecutive CHB patients from the Ruian people’s hospital, Wenzhou, Zhejiang, China, between July 2013 and July 2015, were retrospectively analyzed. Inclusion criteria: (1) the persistent presence of serum HBsAg for more than 6 months; (2) ATL ≤ 2 ULN (the ULN is 40 IU/L); (3) had liver biopsy, LSM, and routine laboratory tests. Exclusion criteria: (1) significant alcohol consumption (>20 g/day) (n = 24); (2) co-infection with HCV or HDV (n = 10); (3) accompanied with autoimmune liver disease (ALD) or nonalcoholic fatty liver disease (NAFLD) (n = 14); (4) prior or current antiviral therapy (n = 32); (5) body mass index (BMI) >28 kg/m^2^ (n = 13); (6) inappropriate biopsy samples (n = 8); (7) unreliable LSM values (n = 16). Finally, 188 patients were included in this study. Figure 1 summarized the flow diagram of the study population.

All patients signed the informed consent before liver biopsy, and all clinical procedures were in accordance with the Helsinki declaration. The ethics committee of Ruian people’s hospital approved the study protocol. All experiments were performed in accordance with relevant guidelines and regulations^13,14^.

### Liver histological assessment

Percutaneous liver biopsy was performed. Liver samples were fixed in 10% formalin, embedded in paraffin, and stained with hematoxylin and eosin. A minimum of 15 mm of liver tissue with at least 6 portal tracts was considered suitable for histological scoring^14^. The biopsy samples were assessed by two independent pathologists blinded to the results of non-invasive fibrosis tests. Discordant cases were reviewed by a third highly experienced liver pathologist. The METAVIR scoring system was used to determine liver fibrosis grade^15^: F0, no fibrosis; F1, portal fibrosis without septa; F2, portal fibrosis with rare septa; F3, numerous septa without cirrhosis; and F4, cirrhosis. We defined significant fibrosis as F2-F4, severe fibrosis as F3-F4, and cirrhosis as F4.

### Liver stiffness measurement

LSM was performed by operators trained according to the manufacturers’ recommendations using FibroScan (Echosens; Paris, France) equipped with the M probe (3.5 MHz transducer, measurement of liver stiffness take place between 25 and 65 mm) within one week of liver biopsy. Briefly, LSM was performed following an overnight period of fasting. Mild amplitude and low-frequency vibrations were transmitted to the liver of each patient, inducing an elastic shear wave propagating through the underlying liver tissue. The velocity of the wave directly correlated to the tissue stiffness. The LSM values may be considered reliable when 10 valid measurements are obtained, with a success rate of ≥60% and an interquartile range/median LSM ≤30%^16,17^.

### Routine laboratory tests

Fasting blood samples were obtained, and routine laboratory tests were performed within one week of liver biopsy. Serum HBsAg was detected using the enzyme-linked immunosorbent assay kit (Wanti BioPharm, Inc., Beijing, China). Serum HBV DNA was measured using the kit for PCR (ABI 7500; Applied Biosystems, Foster City, USA) with a limit detection of 500 copies/ml. Serum biochemical parameters including ALT were measured using full automated biochemistry analyzer (AU2700; Olympus Corporation, Tokyo, Japan).

### Serum fibrosis models calculation

1. APRI = (AST (IU/L)/ULN of AST)/platelet count (10^9^/L) × 100; ULN of AST = 40 IU/L.
2. FIB-4 = (age (years) × AST (IU/L))/(platelet count (10^9^/L) × (ALT (IU/L))^1/2^).

### Statistical analysis

The Kolmogorov-Smirnov test was used to verify the normal assumption of quantitative data. The baseline data was presented as follows: normal distribution data as mean ± standard deviation, non-normal distribution continuous data as median (interquartile range (IQR)), and categorical variables as number (percentage). The t-test (for normal distribution variables), Mann-Whitney test (for non-normal distribution continuous variables), and Chi-squared test (for categorical variables), respectively, were performed to identify the statistical differences between two groups. The correlation analysis was performed using the Spearman test. The diagnostic performance was assessed using the receiver operating characteristic (ROC) curves. The area under ROC curves (AUROCs) were compared using Z-test^18^. The optimal cut-off was obtained by maximizing Youden index (sensitivity + specificity-1). Diagnostic performance was evaluated by sensitivity, specificity, positive predictive value (PPV), negative predictive value (NPV), positive likelihood ratio (PLR), negative likelihood ratio (NLR), and diagnostic accuracy (DA). All significance tests were two tailed, and *p* < 0.05 was considered statistically significant. All statistical analyses were carried out using SPSS statistical software version 15.0 (SPSS Inc. Chicago, IL, USA) and MedCalc Statistical Software version 16.1 (MedCalc Software bvba, Ostend, Belgium).

## Results

### Baseline characteristics of patients

The baseline characteristics of patients were shown in Table 1. The majority of patients were male (62.8%), HBeAg positive (66.0%), and middle-aged (37 ± 10 years). The median BMI, HBV DNA, AST, GGT, total bilirubin, and albumin were 22.5 kg/m2 (IQR = 20.4–24.2), 5.4 log10 copies/ml (IQR = 3.2–7.5), 26 IU/L (IQR = 21–34), 17 IU/L (IQR = 13–29), 13 μmol/L (IQR = 10–17), and 46 g/L (IQR = 44–48), respectively. The mean ALT and platelet count levels were 39 IU/L and 196 × 10^9^/L, respectively. The Median LSM value, APRI, and FIB-4 scores were 5.8 kPa (IQR = 3.7–8.5), 0.40 (IQR = 0.29–0.60), and 1.0 (IQR = 0.7–1.5), respectively. The METAVIR inflammation grades were as follows: A0 = 17 (9%); A1 = 76 (40.4%); A2 = 61 (32.4%); and A3 = 34 (18.9%). The METAVIR fibrosis grades were as follows: F0 = 11 (5.9%); F1 = 83 (44.1%); F2 = 54 (28.7%); F3 = 12 (6.4%); and F4 = 28 (14.9%).

**Table 1:** Characteristics of the study subjects.

Characteristic	All patients (n = 188)	F0-F1 (n = 94)	F2-F4 (n = 94)	p Value
Male sex (n, %)	118 (62.8%)	59 (62.8%)	59 (62.8%)	
Age (years)	37 ± 10	34 ± 9	40 ± 10	<0.001
BMI (kg/m2)	22.5 (20.4–24.2)	22.3 (20.1–23.8)	22.6 (20.8–24.6)	0.962
HBeAg positive (n, %)	124 (66.0%)	37 (39.4%)	87 (60.6%)	<0.001
HBV-DNA (log10 copies/ml)	5.4 (3.2–7.5)	3.2 (2.7–4.1)	7.5 (4.9–7.7)	<0.001
ALT (IU/L)	39 ± 16	35 ± 16	44 ± 16	<0.001
AST (IU/L)	26 (21–34)	25 (21–31)	28 (23–37)	0.003
GGT (IU/L)	17 (13–29)	19 (13–34)	16 (13–26)	0.173
Platelet count (10^9^/L)	196 ± 52	197 ± 54	196 ± 51	0.884
Total Bilirubin (μmol/L)	13 (10–17)	14 (10–17)	13 (10–17)	0.260
Albumin (g/L)	46 (44–48)	47 (44–48)	46 (44–48)	0.418
LSM value (kPa)	5.8 (3.7–8.5)	4.2 (2.8–5.5)	8.4 (6.6–10.8)	<0.001
APRI	0.40 (0.29–0.60)	0.33 (0.27–0.42)	0.50 (0.34–0.76)	<0.001
FIB-4	1.0 (0.7–1.5)	0.9 (0.6–1.3)	1.1 (0.7–2.1)	0.010
METAVIR inflammation grade (A0/A1/A2/A3)	17 (9%)/76 (40.4%)/61 (32.4%)/34 (18.9%)			
METAVIR fibrosis grade (F0/F1/F2/F3/F4)	11 (5.9%)/83 (44.1%)/54 (28.7%)/12 (6.4%)/28 (14.9%)			

BMI, body mass index; ALT, alanine transaminase; AST, aspartate transaminase; GGT, gamma-glutamyl transpeptidase; LSM, liver stiffness measurement; APRI, AST to platelet ratio index; FIB-4, fibrosis index based on the 4 factors.

Of 188 patients, 94 (50%) have F2-F4, 40 (21.3%) have F3-F4, and 28 (14.9%) have F4, respectively. Patients with F2-F4 had higher age (40 *vs* 34 years, *p* < 0.001), proportion of HBeAg-positive (60.6% *vs* 39.4%, *p* < 0.001), HBV DNA (7.5 *vs* 3.2 log10 copies/mL, *p* < 0.001), ALT (44 *vs* 35 IU/L, *p* < 0.001), AST (28 *vs* 25 IU/L, *p* = 0.003), LSM value (8.4 *vs* 4.2 kPa, *p* < 0.001), APRI (0.50 *vs* 0.33, *p* < 0.001), and FIB-4 (1.1 *vs* 0.9, *p* = 0.010) than patients with F0-F1. No significant differences were seen in sex, BMI, GGT, platelet count, total bilirubin, and albumin.

### Correlation between noninvasive fibrosis tests and METAVIR fibrosis stages

**Figure 2:**
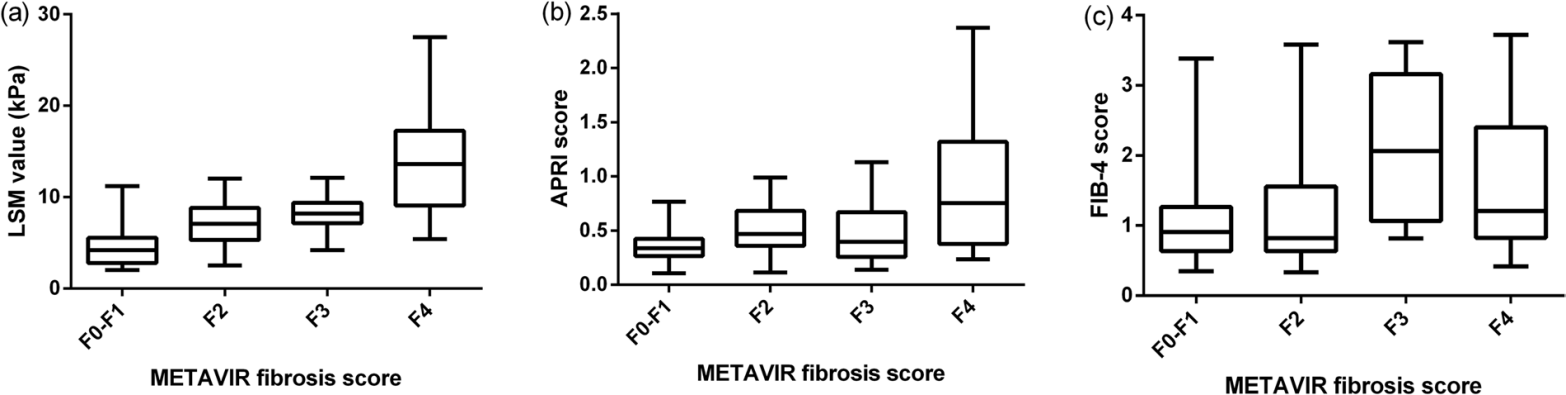
Association between METAVIR fibrosis scores and noninvasive fibrosis tests. LSM, liver stiffness measurement; APRI, aspartate transaminase to platelet ratio index; FIB-4, fibrosis index based on the 4 factors.

The association between METAVIR fibrosis stages and noninvasive fibrosis tests was presented in Table 2 and Fig. 2. The METAVIR fibrosis stages were positive correlated with LSM values (r = 0.72, *p* < 0.001), APRI (r = 0.43, *p* < 0.001), and FIB-4 (r = 0.27, *p* < 0.001). LSM values, APRI, and FIB-4 tended to increase with the increased METAVIR fibrosis stages (Fig. 2).

**Table 2:** Correlations between noninvasive fibrosis tests and METAVIR fibrosis stages.

Variables	Spearman’s r	p value
LSM	0.72	**<0.001**
APRI	0.43	**<0.001**
FIB-4	0.27	**<0.001**

LSM, liver stiffness measurement; Spearman’s r, correlation coefficient.

### Diagnostic performances of noninvasive fibrosis tests

**Figure 3:**
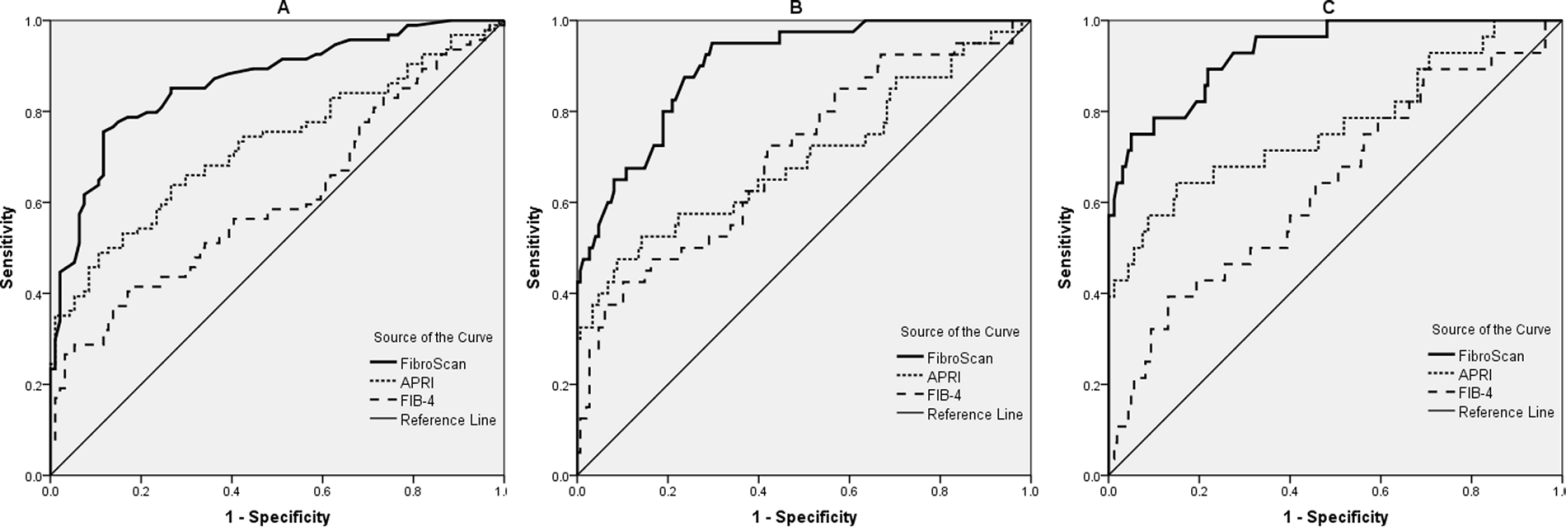
ROC curves for significant fibrosis (**A**), severe fibrosis (**B**), and cirrhosis (**C**). APRI, aspartate transaminase to platelet ratio index; FIB-4, fibrosis index based on the 4 factors.

The ROC curves of noninvasive fibrosis tests were shown in Fig. 3. The AUROCs of noninvasive fibrosis tests were shown in Table 3. To predict F2-F4, the AUROC of LSM was higher than that of APRI (0.86 *vs* 0.73, *p* = 0.001) and FIB-4 (0.86 *vs* 0.61, *p* < 0.001). To predict F3-F4, the AUROC of LSM was also higher than that of APRI (0.90 *vs* 0.70, *p* < 0.001) and FIB-4 (0.90 *vs* 0.70, *p* < 0.001). To predict F4, the AUROC of LSM was significantly higher than that of APRI (0.93 *vs* 0.77, *p* = 0.012) and FIB-4 (0.93 *vs* 0.64, *p* < 0.001).

**Table 3:** The AUROCs for noninvasive fibrosis tests in predicting liver fibrosis and cirrhosis.

	Significant fibrosis		Severe fibrosis		Cirrhosis	
	AUROC	(95% CI)	AUROC	(95% CI)	AUROC	(95% CI)
FibroScan	0.86	(0.81–0.91)	0.90	(0.85–0.94)	0. 93	(0.89–0.96)
APRI	0.73	(0.66–0.79)	0.70	(0.63–0.76)	0.77	(0.70–0.83)
FIB-4	0.61	(0.53–0.68)	0.70	(0.63–0.77)	0.64	(0.56–0.71)
AUROC Comparison						
FibroScan and APRI	*p* = *0.001*		*p* < *0.001*		*p* = 0.012	
FibroScan and FIB-4	*p* < *0.001*		*p* < *0.001*		*p* < *0.001*	
APRI and FIB-4	*p* = *0.009*		*p* = 0.926		*p* = 0.015	

AUROC, area under the receiver operating characteristic curve; CI, confidence interval; Significant fibrosis, METAVIR F2-F4; Severe fibrosis, METAVIR F3-F4; Cirrhosis, METAVIR F4.

### Diagnostic thresholds of noninvasive fibrosis tests

The diagnostic thresholds of noninvasive fibrosis tests were presented in Table 4. Maximizing Youden index, the cut-off values of LSM were 6.5 kPa, 7.4 kPa, and 9.5 kPa, for predicting F2-F4, F3-F4, and F4, respectively. The cut-offs of APRI were 0.52 for predicting F2-F4, and 0.62 for predicting F4. The optimal cut-off of FIB-4 was 1.8 for predicting F3-F4.

**Table 4:** Diagnostic thresholds of noninvasive fibrosis tests.

	Cut-offs	Se (%)	Sp (%)	PPV (%)	NPV (%)	PLR	NLR	DA (%)
**LSM value (kPa)**								
F2-F4	≥6.5	76	88	87	78	6.45	0.28	81
F3-F4	≥7.4	88	76	50	96	3.70	0.16	78
=F4	≥9.5	75	95	72	96	15	0.26	89
**APRI**								
F2-F4	≥0.52	50	89	82	64	4.6	0.57	69
=F4	≥0.62	64	85	43	93	4.29	0.42	81
**FIB-4**								
F3-F4	≥1.80	46	90	53	85	4.19	0.64	79

Se, sensitivity; Sp, specificity; PPV, positive predictive value; NPV, negative predictive value; PLR, positive likelihood ratio; NLR, negative likelihood ratio; DA, diagnostic accuracy; LSM, liver stiffness measurement; Significant fibrosis, METAVIR F2-F4; Severe fibrosis, METAVIR F3-F4; Cirrhosis, METAVIR F4.

### The impact of ALT levels on the diagnostic performances and cutoff values of LSM

To assess the impact of ALT levels on LSM, we stratified the 188 patients into two categories: 107 patients had normal ALT levels, and 81 patients had mildly elevated ALT levels (ULN < ALT ≤ 2 ULN) (Table 5). For predicting significant fibrosis, the AUROC of LSM was 0.86 in patients with normal ALT levels, and 0.84 in patients with mildly elevated ALT levels. For predicting cirrhosis, the AUROC of LSM was 0.88 in patients with normal ALT levels, and 0.98 in patients with mildly elevated ALT levels.

**Table 5:** The AUROCs and optimal cut-offs for LSM according to ALT levels.

	ALT levels	AUROC (95% CI)	Cut off (kPa)	Se (%)	Sp (%)	PPV (%)	NPV (%)	PLR	NLR	DA (%)
Significant fibrosis	≤1 ULN (n = 107)	0.86 (0.78–0.92)	6	83	88	81	89	6.91	0.20	82
	1–2 ULN (n = 81)	0.84 (0.74–0.91)	6.5	74	89	93	63	6.67	0.29	
Cirrhosis	≤1 ULN (n = 107)	0.88 (0.80–0.93)	7.8	75	82	35	96	4.19	0.30	86
	1–2 ULN (n = 81)	0.98 (0.93–1.00)	10.2	94	95	83	98	20.31	0.07	

AUROC, area under the receiver operating characteristic curve; LSM, liver stiffness measurement; ALT, alanine transaminase; CI, confidence interval; Se, sensitivity; Sp, specificity; PPV, positive predictive value; NPV, negative predictive value; PLR, positive likelihood ratio; NLR, negative likelihood ratio; DA, diagnostic accuracy; Significant fibrosis, METAVIR F2-F4; Cirrhosis, METAVIR F4.

Patients with mildly elevated ALT levels had higher cut-off values than patients with normal ALT levels for predicting F2-F4 (6.5 *vs* 6 kPa) and F4 (10.2 *vs* 7.8 kPa). Using cut-offs regardless of ALT levels, the diagnostic accuracy of LSM was 81% for F2-F4, and 89% for F4 (Table 4). Applying ALT-stratified cut-off values, the diagnostic accuracy of LSM was 82% for predicting F2-F4, and 86% for predicting F4 (Table 5).

## Discussion

In this study, we compared the diagnostic performance of LSM with that of serum fibrosis models (APRI and FIB-4). LSM showed significantly higher diagnostic performance than APRI and FIB-4 for the diagnosis of significant fibrosis, severe fibrosis, and cirrhosis. Previous studies had also evaluated the diagnostic performance of LSM in Chinese chronic HBV-infected patients with normal or mildly elevated ALT levels, yet none has compared LSM with serum fibrosis models^19–21^. The advantages of this study include comparison with serum fibrosis models and using liver biopsy as reference.

We confirmed the good performance of LSM to predict significant fibrosis with an AUROC of 0.86, and predict cirrhosis with an AUROC of 0.93 in Chinese CHB patients with ALT ≤ 2 ULN. The results are consistent with European studies^22,23^. A prospective study included 202 CHB patients found that the AUROC of LSM is 0.81 for predicting significant fibrosis, and 0.93 for predicting cirrhosis^22^. Another study included 125 European patients found that the AUROC of LSM is 0.85 for predicting significant fibrosis, and 0.90 for predicting cirrhosis^23^. In a Korea study, the diagnostic performances of LSM were better than our results, with AUROC of 0.94 for predicting significant fibrosis, and 0.96 for predicting cirrhosis^24^. The different histological scoring systems between this study (METAVIR scoring systems) and the Korea study (Batts scoring system) might be a reason for the difference.

The optimal cutoff values of LSM in this study (6.5 kPa for significant fibrosis and 9.5 kPa for cirrhosis) were lower than that reported by Marcellin *et al*. (7.2 kPa for significant fibrosis and 11 kPa for cirrhosis)^22^, and Jia *et al*. (7.3 kPa for significant fibrosis and 10.7 kPa for cirrhosis)^25^. A meta-analysis found that the optimal cutoff values of LSM were 7.9 kPa for significant fibrosis and 11.7 kPa for cirrhosis^26^. Obviously, the LSM cutoff values in this study were lower than previous studies. Three possible reasons are as follows. First, this study was performed in patients with ALT ≤ 2 ULN, while previous studies were performed in general CHB patients including ALT > 2 ULN. As elevated ALT levels were associated with higher LSM value, the cutoff values of LSM in patients with ALT ≤ 2 ULN were lower than general patients including ALT > 2 ULN. Second, the LSM test can be biased by high levels of liver inflammation (ALT > 2 ULN) rather than normal or mildly liver inflammation (ALT ≤ 2 ULN). Moreover, the differences in prevalence of significant fibrosis and cirrhosis might be the third explanation for the reason why the cut-offs presented by this study were not in line with the previously published data^22,25^.

Several studies have showed the impact of ALT levels on LSM value. Chan *et al*. founded that elevated ALT levels were associated with higher LSM value (r = 2.8, *p* < 0.001), and proposed various optimal cut-offs depending on magnitude of ALT elevation^19^. Arena *et al*. also found a positive correlation between ALT levels and LSM values at the onset of acute viral hepatitis (r = 0.53, *p* = 0.02)^27^. Wong *et al*. suggested that serum ALT levels should always be taken into account when interpreting results from LSM, especially in patients who might have HBV flares^28^. However, applying ALT-related cut-off values did not improve the diagnostic accuracy of LSM in this study. Our results is well-supported by other studies^29,30^. Cardoso *et al*. have first challenged the approach of using ALT guided cut-offs for LSM in patients with CHB^29^. Although Cardoso *et al*. found a positive correlation between ALT levels and LSM values (r = 0.365, *p* < 0.001), ALT specific cut-offs did not enhance diagnostic performance in patients with CHB^29^. Seo *et al*. also found that mildly elevated ALT levels did not influence the diagnostic performance of LSM^30^. We concluded that LSM mainly was influenced by acute viral hepatitis, HBV flares, or significantly elevated ALT levels (ALT > 2 ULN) rather than mildly elevated ALT levels.

For predicting cirrhosis, the AUROC of LSM in patients with mildly elevated ALT levels is higher than that in patients with normal ALT levels (0.98 vs 0.88). The difference may be related to difference in cirrhosis prevalence in the studied populations, known as the spectrum bias^31,32^. In this study, the prevalence of cirrhosis in patients with mildly elevated ALT levels is higher than patients with normal ALT levels (19.6% *vs* 11.2%).

Based on evidence from the systematic review, the WHO guidelines recommended that LSM and APRI were the most useful tests for the assessment of cirrhosis in resource-limited settings^33^. Although APRI had been recommended for the assessment of cirrhosis, our results suggest that APRI and FIB-4 are significantly inferior to LSM. Based on our results, we recommended that LSM should be considered as the preferred noninvasive fibrosis tests, and APRI should be considered when LSM is unavailable. Liver biopsy remains within the armamentarium of hepatologists when there are discordances between clinical symptoms and the extent of fibrosis assessed by non-invasive approaches.

This study has several limitations. First, the retrospective analysis might have caused selective bias resulting in underestimated sensitivity and overestimated specificity of non-invasive fibrosis diagnostic models. Second, this study might be not timely. This cohort included 188 patients who had liver biopsies and LSM values between July 2013 and July 2015. Data since July 2015 were lacking.

In conclusion, our study assessed the accuracy of LSM for predicting significant fibrosis and cirrhosis in Chinese CHB patients with ALT ≤ 2 ULN. LSM showed higher diagnostic performances than APRI and FIB-4. For patients with ALT ≤ 2 ULN, ALT levels did not affect the diagnostic performance of LSM, and ALT-stratified cut-off values did not enhance diagnostic accuracy of LSM in this specific population.
